# Irrelevant angry faces impair response inhibition, and the go and stop processes share attentional resources

**DOI:** 10.1038/s41598-022-19116-5

**Published:** 2022-10-10

**Authors:** Shubham Pandey, Rashmi Gupta

**Affiliations:** Cognitive and Behavioural Neuroscience Laboratory, Department of Humanities and Social Sciences, Indian Institute of Technology Bombay, Mumbai, Maharashtra 400076 India

**Keywords:** Neuroscience, Psychology

## Abstract

Response inhibition is a crucial component of executive control, which refers to our ability to suppress responses that are no longer needed or inappropriate. The stop-signal task is a standard tool to assess inhibitory control over actions. Here, we use irrelevant facial expressions (happy, angry, or neutral) as both go and stop-signal to examine competition for shared attentional resources between (a) emotion and inhibition process and (b) go and stop processes. Participants were required to respond to go signals (gender discrimination task: male or female). Occasionally, a stop-signal (face with irrelevant angry, happy, or neutral facial expression) was presented, and participants were required to withhold their motor response. We found that emotion processing (especially angry faces) captures attention away from the task, and the emotionality of the stop signal matters only when the go signal is non-emotional. When the go signal was non-emotional, we found that stop-signal with irrelevant angry facial expressions impaired inhibitory control compared to stop-signal with irrelevant happy and neutral facial expressions. These results indicate that the processing of emotion and inhibition process exploit a shared pool of attentional resources. These results favor an interactive capacity-sharing account of the go and stop processes in models of response inhibition.

## Introduction

Emotional information (e.g., a smiling face) plays a crucial role in daily life. Processing emotional information receives attention prioritization^1–5^, therefore, it affects cognition and behaviour. Maladaptive processing of emotional information can lead to various psychopathologies (e.g., depression, anxiety, ADHD, personality disorder, etc.)^6–9^. Therefore, studying how emotions interact with various cognitive processes (e.g., attention, perception) is crucial. Here, we investigate the effect of faces with irrelevant emotional information on response inhibition. We show that stop signals with irrelevant facial expressions affect response inhibition only when the go signal has non-emotional information.

### Emotion and attention

Previous research indicates that emotion processing (specifically happy and angry/sad faces) interacts with attention differently. For example, the processing of happy faces distributes the scope of attention and the processing of angry/sad emotions narrows or focuses the scope of attention^10–15^. Also, processing happy faces requires fewer attentional resources^16^. In contrast, processing angry faces takes a lot of attentional resources^14,17,18^. Together, these results indicate that angry and happy faces capture attention differently.

### Response inhibition, attention, and emotion

Response inhibition involves suppressing an initially planned prepotent response^19^. There are many examples of the importance of response inhibition in our day-to-day lives, such as refraining from crossing a road when a car suddenly comes around the corner. In the laboratory, the stop-signal task has been frequently used to study response inhibition^19^. In this task, participants respond to a go signal on most trials and refrain from responding when presented with a stop signal on infrequent trials. Performance on stop-signal task has been modelled as a race between a go process and a stop process independently rising to a threshold (Race model^19,20^). Whichever process reaches the threshold first, that response is executed. However, neurophysiological data argue against complete independence between the go and stop processes^21^⁠. The cancellable-rise-to-threshold (CRTT) model, an alternative to the race model, does not assume independence of the go and the stop processes. Instead, it advocates an interactive capacity-sharing account of the go and stop processes where the go process is decelerated by rapid sensory detection of the stop signal^22–24^. In the present study, we investigated this capacity sharing account of the go and stop processes by simultaneously manipulating the irrelevant emotional information of go and stop signals. It has been suggested that emotional information and attentional resources interact^18^. Therefore, simultaneous manipulation of irrelevant emotional information in both signals (go and stop) would help understand the interactive role of go and stop processes in response inhibition.

In line with this view, it has been suggested that successful inhibition depends on attentional resources' availability (see the executive act of control model of Logan & Cowan, 1984^20^). Attention serves as an executive giving orders to subordinate systems with its selective, controlling influence. If emotional information captures or diverts the allocation of attention away from main task demands such as inhibition, emotional information should interfere with response inhibition. Previous studies have shown that enhanced emotion processing^25^ competes for attentional resources with the inhibition process. Pessoa (2009)^26^ formalized this competition into a “dual competition framework” that posited that executive control sub-components mutually interact with each other such that resources utilized by one component will not be available to other components^26^. Hence, according to this framework, processing faces with irrelevant emotional information would consume a major chunk of available resources leaving fewer processing resources available to inhibit preplanned response; thus, face stimuli with irrelevant emotional information, in general, would impair inhibitory control. Studies have found impaired inhibitory control for emotional distractors (positive and negative) compared to neutral distractors^27–29^ and for negative distractors compared to neutral^29–31^. These studies used emotional stimuli as prime (before the trial) instead of manipulating the irrelevant emotional information (implicit emotion) of go or stop signal; it has been argued that prime emotion has less effect on cognition; implicit emotion has a strong effect^32^.

Very few studies have manipulated the irrelevant emotional information of face stop-signal^33–35^. Studies have shown that negative faces, such as fearful faces, impair response inhibition compared to happy faces^35^. However, other studies indicate that irrelevant happy and fearful faces facilitate inhibition compared to neutral faces^33^, and happy faces facilitate inhibition compared to neutral faces^36^. Thus, the results are mixed when using emotional face stimuli as a stop signal. Studies using IAPS images found that negative images impair response inhibition^30,37^. In contrast, Verbruggen and De Houwer^38^ suggested that high arousing positive and negative pictures interfered with response inhibition than low arousing positive and negative pictures. These mixed findings might be due to complexity of stimuli and unmatched arousal levels especially in case of IAPS images. Additionally, these studies did not check for the competition of attentional resources between the go and stop processes, which might have affected the pattern of results, especially in those studies where the stop signal was presented by manipulating the frame color of the go face. Therefore, presenting emotional information in both go and stop-signal is crucial to understanding the nature of shared attentional resources and ascertaining previous results.

In the current study, we use grayscale face stimuli. Most of the previous studies used IAPS pictures. Notably, IAPS pictures are complex. Also, there are low-level visual differences between positive and negative pictures (e.g., negative images such as mutilated bodies are dominated by red color, whereas the skin color dominates positive images such as erotic pictures). It has been suggested that emotional faces vary less on low-level features than IAPS pictures^14,34^. Facial stimuli also have high social and evolutionary value. Hence, in the current study, we use grayscale face stimuli as both go and stop signals.

### The present study

The present study examines the effect of faces with irrelevant emotional information on response inhibition. In previous studies, stimuli with emotional information were mainly used as prime; or studies manipulated emotional information of either the go^28,39^ or stop signals^33,35,36^. None of the studies manipulated the emotional information of both go and stop-signal using the stop-signal task. Therefore, these studies could not examine the competition for shared attentional resources between the go and stop processes. Thus, in the present study, we introduced faces with irrelevant emotional information as both the go and stop signal to examine the competition for shared attentional resources between the go and stop processes and, in general, competition between emotional information and inhibition. Since processing angry faces requires a lot of attention resources^14^, we hypothesize that irrelevant angry facial expression of the go signal would impair inhibitory control compared to irrelevant neutral and happy facial expressions of the go signal. As per the “dual competition framework”, we predicted that irrelevant emotional information in the go signal would consume available attentional resources leaving fewer resources for the inhibition process. Hence, when the go signal has irrelevant emotional information, there would be no modulation in inhibitory control due to the emotional information of stop signals as majority of attentional resources would already have been consumed by go signal. However, when the go signal would have no emotional information, i.e., neutral face go signals, then irrelevant emotion of stop signals may modulate response inhibition. Since angry faces take more attentional resources to be processed, we predict that this modulation would be such that stop signals with irrelevant angry facial expressions would impair inhibition compared to stop signals with irrelevant happy facial expressions.

## Method

### Ethics statement

The study was carried out in accordance with the Declaration of Helsinki and was approved by the Institute Ethics Committee of the Indian Institute of Technology Bombay. All participants provided informed consent.

#### Participants

Fifty-six volunteers (30 Male) aged 18–34 years (M = 21.5 years, SD = 3.9 years) with normal or corrected to normal vision were recruited through flyer advertisements and e-mail from the Indian Institute of Technology Bombay personnel. We estimated (using repeated measure within factor F-test in G-Power^40^) a necessary sample size of 45 to detect a medium-size effect of 0.25 and obtain a power level of 0.95. All participants gave written consent to take part in the study. All participants were in good health, free of medications, and had no psychiatric or neurological disease history.

#### Apparatus and stimuli

Participants were seated in a nearly dark room at a distance of ~ 57 cm in front of a 24-inch LCD flat-screen B360 Gaming HD monitor, Intel(R) Core(TM) i7 CPU @3.20 GHz system of resolution 1920 × 1080, scan rate 60 Hz running Microsoft Windows 10 Pro. Visual stimuli were presented with the help of PsychToolbox in MATLAB® (Mathworks Inc).

A total of 36 images of faces (12 identities, three expressions, six females and six males) were selected from the NimStim facial database^41^. These faces were selected based on the accuracy of gender identification and ease of emotion recognition by six volunteers in the pilot session. Faces with 100% correct recognition by all participants were selected for the experiment. These faces were then cropped so that only the face portion was visible without hair, neck, and ears. The cropped faces were then converted into grayscale images with the help of GIMP software. Then these 36 faces were grouped into two groups (18 faces, six identities). Later in the experiment, half of the participants got the first group faces as go faces and the second group faces as stop-signal faces while the other half vice-versa; therefore, faces used for go signal and stop signal were counterbalanced across participants.

#### Experimental procedure

**Figure 1:**
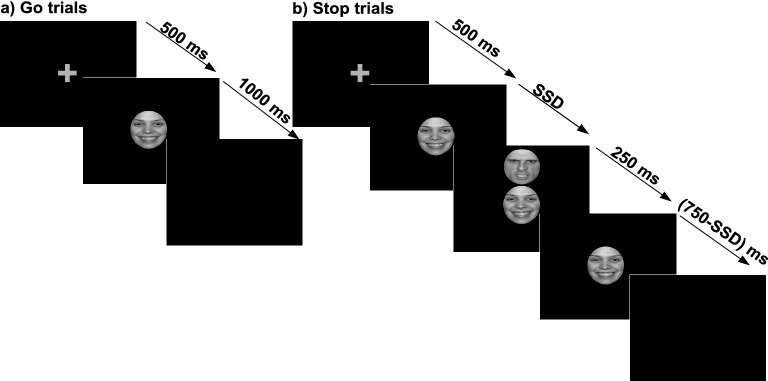
*Schematic of Emotional Stop Signal Task.* An example of a go and a stop trial. (**a**) During go trials, participants were required to press the left arrow key for male faces and the right arrow key for female faces. (**b**) During stop-trials, they were required to withhold their motor response (signaled by a face at the top of go face with irrelevant happy, angry, or neutral emotions). The stop-signal followed the go-signal after a variable time delay called stop-signal delay (SSD). The SSD was set based on a staircase procedure separately for each stop-signal condition to get stop-performance at approximately 50% correct.

The trial began with the gray plus sign fixation (0.25° × 0.25°) at the center of the screen for 500 ms. In the go trials, an emotional or neutral face appeared at the center of the screen (go-signal) (Fig. 1a). Participants were instructed to press the left arrow key with the index finger for a male face and the right arrow key with the middle finger for a female face. The go-signal stayed there for 1000 ms irrespective of participants’ responses^34,35,42–44^. A blank screen followed this for a variable inter-trial-interval ranging from 500 to 1500 ms drawn from a Gaussian distribution, and then the next trial started.

Stop-signals were presented on 30% of total trials. In these trials, after fixation and go-signal, another face (250 ms duration) appeared at the top of the go-signal (Fig. 1b). This instructed participants not to press any button. The delay between go signal and stop-signal onset is called stop-signal delay (SSD). The initial value of the SSD was set to 250 ms. The SSD was adjusted dynamically throughout the experiment. If participants successfully inhibited their response on a stop trial, the SSD was increased by 50 ms on the subsequent stop trial. The SSD was reduced by 50 ms on the next stop trial if they failed to inhibit their response on previous trial. Since stop-signal had three emotional expressions, this staircase was done separately for three stop-signal conditions to ensure successful inhibition on approximately 50% of the stop trials. Three go signals with three stop signals yielded a total of nine conditions.

There were total six blocks. Each block had 180 trials, 70% go trials (126 trials, 42 trials of angry, happy, and neutral faces each), and 30% stop trials (54 trials, 18 trials of angry, happy, and neutral faces each; six-stop trials of each of the nine conditions). A stop-trial was always followed by a go trial. In go trials, if the participants did not press any button or pressed the button too late after passing a window of 1000 ms from go stimulus onset, an omission error (OE) occurred. If participants pressed the wrong button, a discrimination error (DE) occurred. On stop trials, participants needed not to press. A commission error (CE) occurred if they still pressed a button. To prevent participants from developing a strategy of waiting, we used two strategies; first, the maximum allowed time for response was set to 1000 ms. Second, as per Verbruggen et al^45^ recommendations, participants were shown feedback of their performance on the inter-block window at the end of each block, including omission, discrimination, and commission errors. Participants were instructed to respond as quickly and accurately as possible as per the recommendations made by Verbruggen et al^45^. They were also told that sometimes it might not be possible to inhibit their response successfully and that, in such cases, they should continue performing the task. Overall, the importance of the go and stop response was stressed equally. A fixed compensation of ₹150 was provided to all participants after the successful completion of the task.

Each participant was provided with an initial 30 trials training session to familiarize them with the task and estimate their omission and discrimination error on the go trials and commission error on stop trials. If errors on go trials were higher than five percent or error on stop trials was higher than 50%, the participant was given another practice session. Two different neutral uncropped grayscale faces from the KDEF database^46^ were used for practice.

#### Study design

The present study was exploratory as this was the first study where the emotion of both go and stop signals were manipulated simultaneously. It employed a within-subject design. The irrelevant emotional information (angry, happy, neutral) in go and stop-signal served as a within-group factor. Performance on go trial was subjected to one-way repeated measures ANOVAs with three factors (go emotion: angry, happy, neutral). Performance on stop-trials was subjected to 3 (go emotion: angry, happy, neutral) × 3 (stop emotion: angry, happy, neutral) repeated measures ANOVAs.

#### Data analysis

Data analysis was performed using an in-house program written in MATLAB® (Mathworks Inc.), and statistical tests were performed using JASP (JASP Team, 2021). To investigate the effect of go emotion, repeated measure analysis of variance (ANOVA) was performed on omission error, discrimination error, and correct go RT for three go emotions. One participant’s overall omission error was very high (16%), and a high inhibition rate (62%). It shows that the participant was not paying attention to the task^45^. Another participant had a neurological injury during childhood. Hence, these two participants were excluded from the rest of the analysis. Following the recommendation of Verbruggen et al^45^, we used the untrimmed go RT distribution for SSRT estimation so that the tracking algorithm would cover the whole distribution of go responses. However, while reporting the correct mean go RT, we removed participant-specific outliers based on 2.5 standard deviations away from the mean.

To investigate sharing of attentional resources between go and stop-process and modulation of inhibitory performance by emotional information of stop-signal, stop-signal reaction time (SSRT), which provides an estimate of the “inhibitory reaction time,” was calculated for all possible nine conditions by subtracting the mean stop-signal delay of that condition from mean go RT of corresponding go emotion as per the procedure of Race model^19,45^. It was done separately for all nine conditions, i.e., angry-angry, angry-neutral, angry-happy; neutral-angry, neutral–neutral, neutral-happy; happy-angry, happy-neutral, happy-happy (referred now onward as AA, AN, AH; NA, NN, NH; HA, HN, HH respectively). Here, in each pair, the first letter is the emotionality of the go-signal, while the second letter is the emotionality of the stop-signal.

We calculated another measure of inhibitory control, ‘the attenuation rate’, based on the CRTT model^23,24^. The CRTT model posits an exponential increase in reaction time on unsuccessful stop trials (noncancelled RT) as parallel processing time (PPT) increases. PPT is the maximum time duration for which the go and stop signal can be processed in parallel before eliciting a response. Empirically, it is calculated by subtracting SSD from noncancelled RT. PPT was grouped from 0 to 400 ms into bins of size four refresh rate duration. The mean PPT and mean noncancelled RT were calculated across trials in each bin. Noncancelled RT was plotted against PPT and fitted with an exponential fit (noncancelled RT = *εe*^*b*(PPT)^ + *c*, see Indrajeet and Ray (2020) for a detailed derivation based on CRTT model^24^) weighted by reciprocal of the standard error of the mean (SEM) of noncancelled RT in each bin. The coefficient *b* in this equation refers to ‘the attenuation rate’. A higher attenuation rate reflects a better ability to decelerate an ongoing motor plan. Per previous guidelines, we fixed ε at 17 ms, i.e., one refresh duration, to account for random jitter in the measurements of RT and SSD^24,47^. Those participants with goodness-of-fit less than the minimum fixed criteria (*R*^*2*^ < 0.5) in any three emotion categories were removed for computation of attenuation rate. Finally, we also compared noncancelled RT on unsuccessful stop-trails to RT on correct go trials to check for any violation of the independence assumption of the race model. Bonferroni correction was applied for post-hoc paired t-tests for discrimination error, omission error, correct go RT, SSRT, and attenuation rate such that the threshold for *p* value for three emotional conditions was adjusted to 0.01 (0.05/3).

## Results

Descriptive and behavioural results are summarized in Table 1.

**Table 1:** All values represent mean (standard deviation).

Descriptive Statistics and Behavioral Results									
Behavioral measure: go trials									
Measure			Go Emotion						
			Angry		Neutral		Happy		
Omission Error (%)			6.16 (3.91)		5.36 (3.4)		5.56 (3.5)		
Discrimination Error (%)			4.36 (3.9)		3.24 (2.4)		3.23 (2.6)		
Correct Go RT (ms)			730.80 (59.8)		723.76 (60.3)		723.55 (63.4)		
Noncancelled RT (ms)			692.35 (67.2)		680.79 (69.3)		681.66 (68.8)		

Behavioral measures: stop-signal trails									
	Condition								
Measure	AA	AN	AH	NA	NN	NH	HA	HN	HH
SSD (ms)	519.05 (91.1)	519.80 (90.4)	522.57 (94.1)	518.16 (90.1)	519.98 (89.7)	526.53 (92.5)	519.30 (89.6)	519.52 (89.2)	522.83 (94.1)
SSRT (ms)	213.66 (51.64)	212.56 (49.68)	210.01 (45.69)	206.51 (49.07)	204.71 (47.26)	198.33 (45.48)	205.34 (47.8)	205.21 (48.76)	201.87 (48.76)
Inhibition Rate (%)	54.47 (6.5)	52.57 (6.1)	55.96 (7.4)	54.16 (7.5)	53.05 (6.9)	51.08 (7.3)	52.31 (7.1)	54.98 (7.2)	53.96 (8.0)

SSD = stop-signal delay; SSRT = stop-signal reaction time; RT = reaction time; Noncancelled RT: RT from those trials where the participant could not withhold their response; AA, AN, AH; NA, NN, NH; HA, HN, HH respectively correspond to angry-angry, angry-neutral, angry-happy; neutral-angry, neutral–neutral, neutral-happy; happy-angry, happy-neutral, happy-happy. Here first emotion is the emotion of the go signal and the second emotion is the emotion of the stop signal.

### Go trials

The result from go trials showed a consistent pattern of higher attentional resource consumption by angry faces compared to happy and neutral faces. Notably, discrimination error and correct go RT were higher for angry faces compared to happy and neutral faces, and omission error was higher for angry faces compared to neutral faces, as discussed below:

*Discrimination error:* The main effect of go emotion on discrimination error was significant, *F*(1, 53) = 6.91, *MSE* = 3.43, *p* = 0.002, *η*_*p*_^*2*^ = 0.11. Paired t-test revealed that discrimination error for angry (M = 4.36%, SD = 3.9%) go faces was significantly higher compared to neutral (M = 3.24%, SD = 2.4%), *t*(53) = 2.96, *p* = 0.005, *d* = 0.40, and happy (M = 3.23%, SD = 2.6%) go faces, *t*(53) = 2.65, *p* = 0.01, *d* = 0.36. There was no significant difference in discrimination error between happy and neutral go faces, *t*(53) = 0.1, *p* = 0.90, *d* = 0.01 (see Table 1).

*Omission error:* The main effect of go emotion on omission error was significant, *F*(2, 106) = 3.70, *MSE* = 2.49, *p* = 0.028, *η*_*p*_^*2*^ = 0.06. Paired t-test revealed that omission error for angry (M = 6.16%, SD = 3.91%) go faces was significantly higher than neutral (M = 5.36%, SD = 3.4%), *t*(53) = 2.78, *p* = 0.007, *d* = 0.37. There was no significant difference in omission error between angry and happy go faces, *t*(53) = 1.90, *p* = 0.062, d = 0.25, and between neutral and happy go faces, *t*(53) = 0.64, *p* = 0.52, d = 0.08 (see Table 1).

*Correct Go Reaction Time*: The main effect of go emotion on correct go RT was significant, *F*(1, 53) = 10.71, *MSE* = 85.69, *p* < 0.001, *η*_*p*_^*2*^ = 0.16. Reaction time was higher for angry go emotion (M = 730.8 ms; SD = 69.8 ms) compared to happy (M = 723.5 ms, SD = 63.4 ms), *t*(53) = 4.13, *p* < 0.001, *d* = 0.12; and neutral go emotion (M = 723.7 ms, SD = 60.3 ms), *t*(53) = 3.49, *p* = 0.001, *d* = 0.12, which suggest that the processing of angry faces took more attentional resources compared to happy and neutral condition. There was no significant difference in RT between happy and neural go emotion, *t*(53) = 0.13, *p* = 0.89, *d* = 0.01, (see Table 1).

### Race model assumption

The race model predicts that the RT on unsuccessful stop-trials (noncancelled RT) should be faster than correct go RT on go trials. If the result deviates, then race model independence assumption is considered to be violated (Recommendation 7: Verbruggen et al^45^⁠). The noncancelled RT (M = 684.76 ms, SD = 66.42 ms) was significantly faster than correct go RT (M = 727.28 ms, SD = 57.56 ms), *F*(1, 53) = 249.13 *MSE* = 549.29, *p* < 0.001, *η*_*p*_^*2*^ = 0.82. This was true for individual emotion category also, angry: *t*(53) = 12.54, *p* < 0.001, *d* = 1.70, neutral: *t*(53) = 14.13 *p* < 0.001, *d* = 1.92, happy: *t*(53) = 12.09, *p* < 0.001, *d* = 1.64. We also manually checked all subjects one by one for any violation. All but one participant followed independence assumption of race model. For participant no 31, the assumption was violated. We performed all analysis after removing this participant. There was no significant change in results. Thus, we have done final analysis including the participant.

### Stop trials

**Figure 2:**
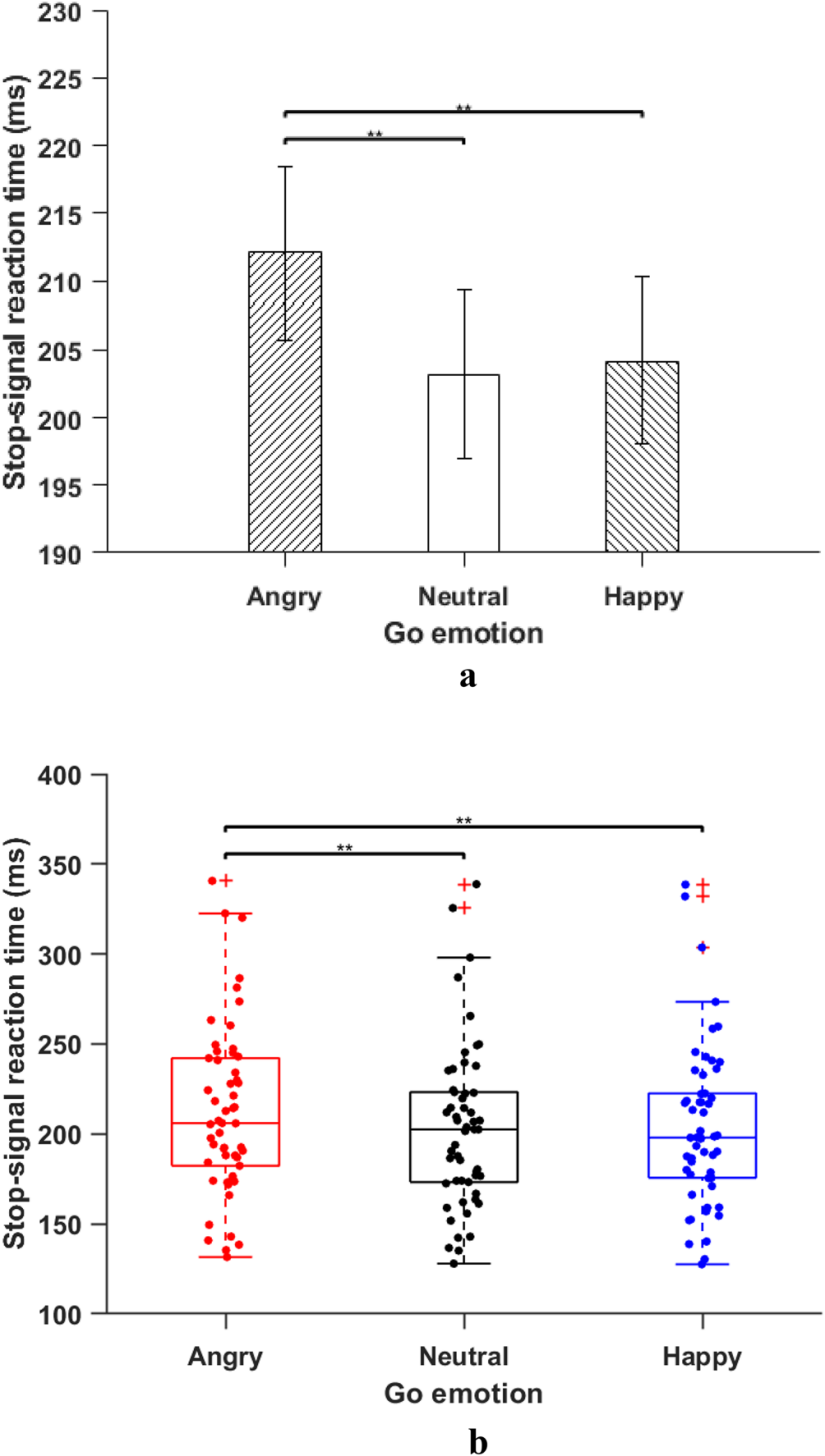
(**a**) *Stop-Signal Reaction Time: Go emotion.* Stop signal reaction time (SSRT) for three go emotions. Error bars indicate the standard error of the corresponding means. The SSRT for the angry go signal was significantly higher than SSRT for the happy and neutral go signal (see text). ^**^ *p* < 0.001. (**b**) *Box-plot distribution of ****a****.* Stop signal reaction time (SSRT) for three go emotions. The SSRT for the angry go signal was significantly higher than SSRT for the happy and neutral go signal (see text). ^**^p < 0.001.

*Stop Signal Reaction Time (SSRT)* The main effect of emotion of go-signal on SSRT was significant, *F*(2, 106) = 11.87, *MSE* = 345.08, *p* < 0.001, *η*_*p*_^*2*^ = 0.17. Pairwise comparisons showed that SSRTs were significantly slower in the presence of irrelevant angry go emotion (M = 212.07 ms, SD = 46.9 ms) compared to neutral (M = 203.18 ms, SD = 45.2 ms), *t*(53) = 4.30, *p* < 0.001, *d* = 0.58, and happy go emotion (M = 204.13 ms, SD = 45.29 ms), *t*(53) = 3.84, *p* < 0.001, *d* = 0.52. This result indicates that irrelevant angry faces as go emotion impaired inhibitory control compared to both neutral and happy go emotion (see Fig. 2a, b for an alternative boxplot). There was no significant difference between SSRTs in the presence of irrelevant happy and neutral go emotion, *t*(53) = 0.46, *p* = 0.096, *d* = 0.06. The main effect of stop emotion was not significant, *F*(2, 106) = 1.54, *MSE* = 762.96, *p* = 0.21, *η*_*p*_^*2*^ = 0.02.

**Figure 3:**
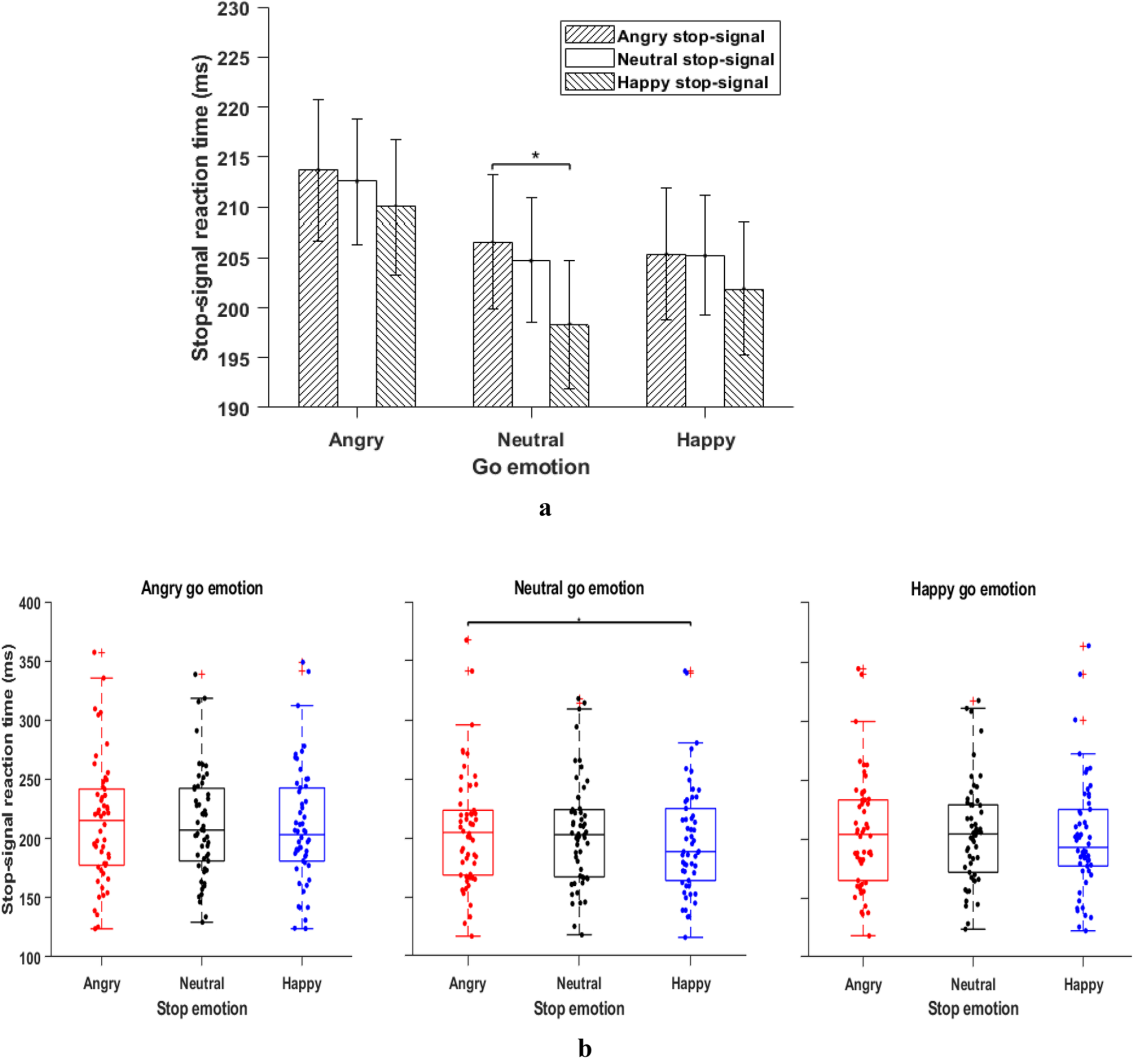
(**a**) *Stop-Signal Reaction Time: Nine Conditions.* Stop signal reaction time (SSRT) for nine conditions grouped as per the emotion of go signal (x-axis). Error bars indicate the standard error of the corresponding means. The legend shows three emotional stop signals. Under neutral go emotion, the SSRT was significantly higher for the angry face stop-signal compared to the happy face stop-signal (see text). ^*^p < 0.05. (**b**) *Box-plot distribution of ****a****.* Stop signal reaction time (SSRT) for stop-signal emotion conditions grouped across three go emotions. Under neutral go emotion, the SSRT was significantly higher for the angry face stop-signal compared to the happy face stop-signal (see text). ^*^p < 0.05.

Figure 3a shows SSRT for nine conditions (Fig. 3b for an alternative boxplot). As predicted, the interaction effect between emotion of go-signal and emotion of stop-signal was significant, *F*(4, 212) = 3.18, *MSE* = 33.68, *p* = 0.014, *η*_*p*_^*2*^ = 0.05, which may indicate that go and stop process interacts with each other. We performed one-way ANOVAs on SSRT score for each go emotion (happy, angry, and neutral) separately. Interestingly, the main effect of stop emotion on SSRT score was only significant when go signal had neutral emotion (neutral go emotion: *F*(2, 106) = 3.59, *MSE* = 277.42, *p* < 0.031, *η*_*p*_^*2*^ = 0.06; happy go emotion: *F*(2, 106) = 0.83, *MSE* = 250.74, *p* = 0.43, *η*_*p*_^*2*^ = 0.01; angry go-emotion: *F*(2, 106) = 0.62, *MSE* = 302.16, *p* = 0.53, *η*_*p*_^*2*^ = 0.01). On neutral go emotion trials, pairwise comparisons indicated that SSRTs were significantly higher for the stop-signal with irrelevant angry facial expression compared to the stop-signal with irrelevant happy facial expression, *t*(53) = 2.60, *p* = 0.012, *d* = 0.35, BF_10_ = 3.13. The neutral-happy comparison (*t*(53) = 1.93, *p* = 0.058, *d* = 0.26, BF_10_ = 0.83) and neutral-angry comparison (*t*(53) = 0.56, *p* = 0.57, *d* = 0.07, BF_10_ = 0.17) were not significant (see Table 2 for all comparisons). Thus, irrelevant angry face stop-signals slow down the response inhibition process compared to irrelevant happy face stop-signal. Thus, angry face as stop-signal impaired inhibition compared to happy face stop-signal when go signal was non-emotional.

**Table 2:** AA, AN, AH; NA, NN, NH; HA, HN, HH respectively correspond to angry-angry, angry-neutral, angry-happy; neutral-angry, neutral–neutral, neutral-happy; happy-angry, happy-neutral, happy-happy. Here first emotion is the emotion of the go signal and the second emotion is the emotion of the stop signal.

Pairwise comparison of stop-signal reaction time (SSRT)					
Pair/Conditions	Mean difference	*t* (53)	*p*	Cohen’s *d*	BF_10_
NA–NN	1.801	0.566	0.574	0.077	0.17
NA–NH	8.179	2.604	0.012*	0.354	3.13
NN–NH	6.378	1.937	0.058	0.264	0.83
AA–AN	1.106	0.332	0.741	0.045	0.15
AA–AH	3.652	1.062	0.293	0.145	0.25
AN–AH	2.547	0.781	0.438	0.106	0.19
HA–HN	0.129	0.047	0.962	0.006	0.14
HA–HH	3.472	1.095	0.279	0.149	0.26
HN–HH	3.343	1.036	0.305	0.141	0.24

*p < 0.05.

### CRTT metric: attenuation rate

**Figure 4:**
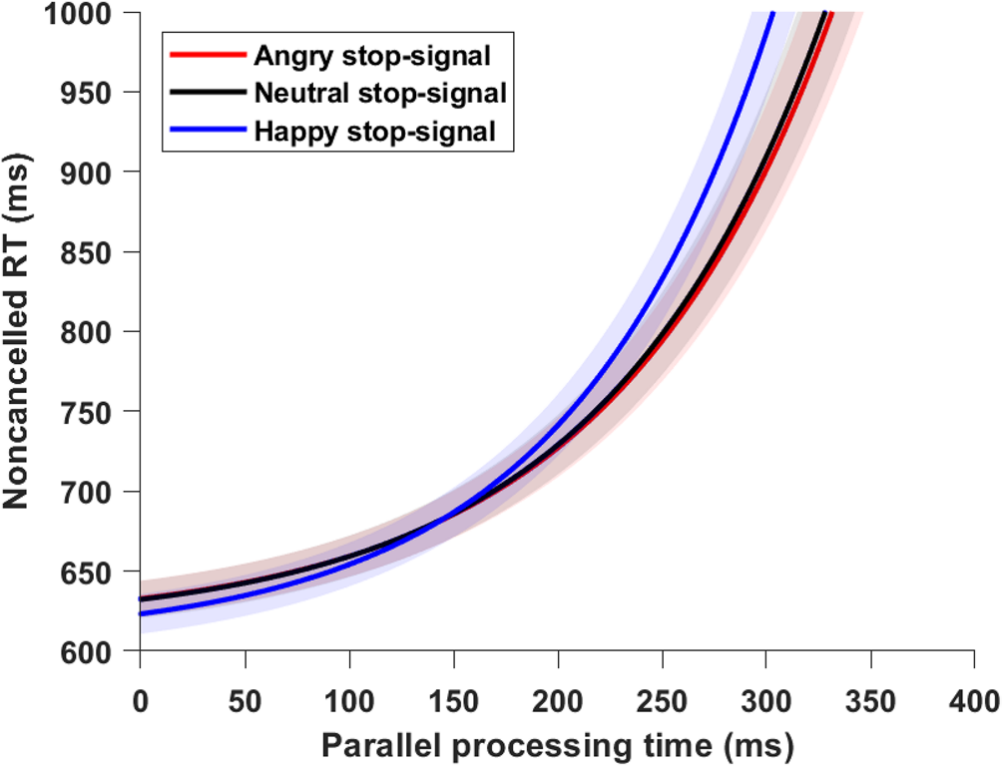
*Noncancelled RT fitted with PPT.* We fitted noncancelled RT and PPT data with an exponential function (noncancelled RT = *εe*^*b(PPT)*^ + *c*). We fixed *ε* at 17 ms (equal to one refresh duration of the display monitor). Coefficients ‘b’ and ‘c’ were fitting coefficients that varied across participants. The average (± SEM) of best fit across participants in three conditions is plotted as a function of PPT. The angry stop signal had the lowest attenuation rate.

**Figure 5:**
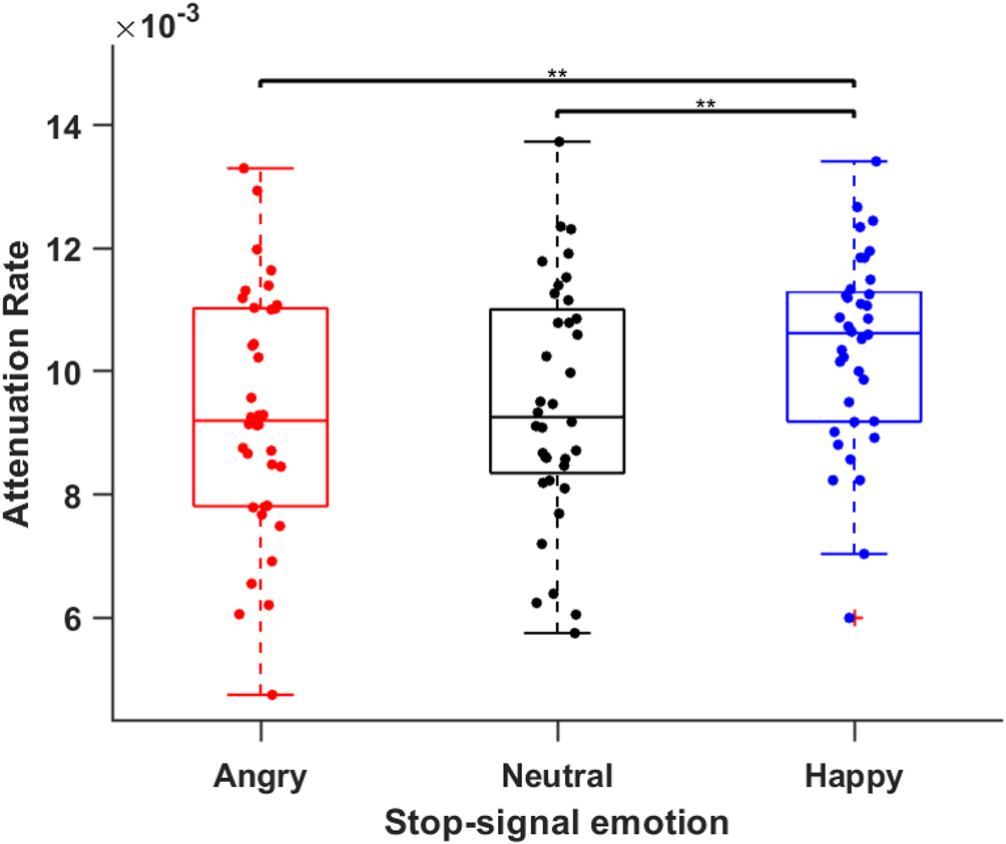
*Attenuation Rate for three Stop-signal.* Attenuation rate, the measure of inhibitory control, is the coefficient *b* in exponential function (noncancelled RT = *εe*^*b(PPT)*^ + *c)*. Boxplot of distributions of attenuation rate for three stop-signal conditions is shown. The average attenuation rate in the angry stop signal condition was lesser than in the happy stop-signal condition, indicating poor inhibitory control with angry faces. ^**^p < 0.01.

The Fig. 4 shows the average of best fit across participants for three stop-signal conditions. The mean goodness-of-fit (*R*^*2*^) was reasonably high: 0.83, 0.89, 0.84 for angry, happy, and neutral stop-signal respectively. A one-way repeated measure ANOVA using emotion of stop-signal (angry, happy, and neutral) as within group factors showed a significant effect of stop emotion on attenuation rate, *F*(2, 70) = 6.52, *MSE* = 0.000001, *p* = 0.003, *η*_*p*_^*2*^ = 0.15 (Fig. 5). Pairwise comparison showed that attenuation rate was lower for angry stop-signal (M = 0.0093; SD = 0.002) compared to happy stop-signal (M = 0.0103, SD = 0.002), *t*(35) = -3.42, *p* < 0.001, *d* = 0.57. It suggests that stop-signal with angry faces exerted less attenuation on go process as processing of angry face consumes more attentional resources leaving less resources for inhibition process. Attenuation rate was lower for neutral stop-signal (M = 0.0094, SD = 0.002) compared to happy stop-signal. There was no significant difference in attenuation rate between angry and neural stop-signal condition, *t*(35) = 0.56, *p* = 0.57.

## Discussion

The present study used irrelevant positive (happy) and negative (angry) face expressions as both go and stop signals compared with the neutral faces to examine the response inhibition. The results indicate that processing irrelevant emotional information from faces and the inhibition process exploits a shared pool of attentional resources. Specifically, angry faces as go and stop signals impair inhibitory control compared to happy faces. Faces with irrelevant emotional information as a go signal also rule out any modulation by the emotionality of the stop signal, i.e., when go signals have irrelevant emotional facial expressions (happy and angry faces), stop latencies in response to stop-signals with happy, angry, and neutral faces do not differ. However, when go-signals have irrelevant neutral facial expressions, stop latencies of stop-signals with irrelevant emotional faces differ. When go signals had irrelevant neutral facial expressions, the stop-signal reaction time (SSRT) was higher for stop-signals with an irrelevant angry facial expression than stop-signals with irrelevant happy facial expressions. It indicates that irrelevant angry faces impair response inhibition compared to irrelevant happy faces. We discuss these results as sharing of resources between emotion and inhibition process and between go and stop processes.

Go signals with irrelevant angry facial expressions impaired inhibitory control compared to go signals with irrelevant neutral and happy facial expressions. Our results showed that angry faces as both go and stop-signal interfere with response inhibition. Previous research suggests that angry faces require a lot of attentional resources compared to happy faces, while happy faces require less attentional resources^17,18^. Our results also show slower reaction time and high discrimination error for go signal with an irrelevant angry facial expression than for go signal with irrelevant neutral and happy facial expression. Angry faces are known to delay the disengagement of attention^48^. Negative emotional information is found to reduce neural activation in brain areas (dorsolateral prefrontal cortex, medial frontal cortex, and parietal cortex) associated with response inhibition^43^, thus, interfering with the inhibition process. A similar study using angry, happy, and neutral faces as go signals found that angry faces impaired inhibition compared to neutral and happy faces^39^. Thus, angry faces consume more attentional resources and interfere with both go and no-go performance.

Our results indicate that stop-signals with irrelevant angry facial expressions impaired response inhibition compared to stop-signals with irrelevant happy facial expressions through the metrics of both race model and CRTT model. Previous studies have shown that negative emotional information impairs inhibitory control^35,49^. A recent study using happy, fearful, and neutral faces as stop-signal showed that fearful faces impaired inhibition compared to happy and neutral faces^35^. However, Pessoa et al. reported that faces with irrelevant fearful and happy facial expressions facilitated response inhibition compared to neutral faces and argued that emotional faces generated enhanced sensory representations of the stop stimulus in the visual cortex, leading to a more robust representation of the stop signal and consequently better stopping performance^33^. This difference in result could be due to a difference in the category of emotional stimuli used. Gupta and Singh^34^ argued that the comparison between happy and fearful faces may not be meaningful/appropriate as both happy and fearful faces produce similar behavioural (approach related), neurochemical (response to oxytocin), and neural responses (amygdala activity) in attention tasks^34^. Thus, comparing happy and angry faces appears more meaningful while studying positive and negative emotions.

Our results do not support the “freezing” behaviour of negative emotions, which predicts that negative emotions should cause a momentary cognitive freeze^50^. Instead, we found that stop-signals with irrelevant angry facial expressions impaired response inhibition compared to stop-signals with irrelevant happy facial expressions. Notice that a freezing account effect would make more sense with evolutionarily significant threatening stimuli (images of spiders, snakes, or electric shock)^51^ as a stop signal. Negative facial expressions like anger, though conveying a potential threat, may not be as threatening as other evolutionarily significant threatening stimuli that carry an immediate danger in the environment and pose a risk to survival (spiders, snakes)^51^. Since we found a valence-specific effect, it rules out the idea that both angry and happy faces generated a more robust representation of the stop signal^33^. However, a stop signal with irrelevant happy facial expressions might have generated a more robust representation of the stop signal than a stop signal with irrelevant angry facial expressions as very few attentional resources are required to process it; therefore, it may slow down the ongoing motor plan compared to stop signals with irrelevant angry facial expressions. It needs to be tested in future studies.

The irrelevant facial expressions of stop signals did not modulate SSRT when the go signal also had irrelevant facial expressions, indicating a common pool of shared attentional resources utilized by the processing of emotion and inhibition process. Our results align with the “dual competition framework”^26^. This framework posits that executive control sub-components mutually interact with each other, such that resources utilized by one component will not be available to other components. Hence, according to this framework, irrelevant emotional information of go signals would already consume most of the available attentional resources leaving fewer processing resources available to cancel preplanned movement on stop trials. In line with this, we found that irrelevant emotions of stop signals did not modulate inhibitory control when the go signals also had irrelevant emotional information. A significant chunk of attentional resources was already consumed by processing the irrelevant emotional information of the go signal. Consistent with this notion, previous magnetoencephalography (MEG) study showed that trials with enhanced go signal processing led to compromised response inhibition, leaving fewer processing resources for the subsequent inhibition^52^. Goldstein et al. reported cognitive-emotional interactions in the dorsolateral prefrontal cortex during a go/no-go task^25^. Thus, the irrelevant emotional information of go signals may compete for attentional resources with the inhibition process. This competition could be due to the finding that neural correlations of emotion processing and response inhibition have been reported in common regions like the anterior cingulate cortex^53^ and right inferior frontal gyrus^54^. Thus, emotion processing and inhibition process exploit a shared pool of attentional resources.

Further, the “dual competition framework” may have implications for the race model of response inhibition by suggesting that both go and stop processes are not entirely independent. Instead, they rely on the same pool of available resources, suggesting an interactive capacity-sharing account of the go process and stop process. In our results, an interaction effect between go emotion and stop emotion on SSRT suggests that the effect of the emotion of stop signal on SSRT is not general; information contained in go signal (go process) and stop-signal (stop process) also plays a key role. Thus, our results show that the factors influencing the go and stop-processes can affect the outcome of the race between the go and the stop processes, and hence, the go and stop processes may not be fully independent. With the CRTT metric, we show that stop-signals with different emotional information decelerate ongoing motor plan (go process) differently, ruling out an independent race between go and stop processes. These results align with recent findings that the go and stop processes are fundamentally inseparable^22,55^. Thus, our results add to the growing literature that go and stop processes share limited central processing capacity^56^ and support interactive race models and interaction between go and stop processes^21,22,24^.

Notably, in the present study, emotional faces were selected from the NimStim database where the actors were African-American, Asian-American, European-American, and Latino-American from different cultural populations, which could be one of the limitations of the present study. Also, the arousal levels of both happy and angry faces were not measured. The current study should be replicated with other face databases to rule out any cross-cultural bias related to emotion processing. Additionally, our aim was not to examine the role of gender and emotional differences in response inhibition; therefore, gender was not matched for go and stop-signal faces. Future studies can examine these two factors together in response inhibition.

## Conclusion

To summarize, we investigated the effect of irrelevant emotional information on response inhibition by employing angry, happy, and neutral faces as both go and stop-signal. We found that the emotional information of the stop signal matters only when the go signal is non-emotional, which indicates the need for sufficient attentional resources for the stop process to modulate inhibition. When attentional resources are more available (in the case of go signals with irrelevant neutral facial expressions compared to go signals with irrelevant happy and angry facial expressions), irrelevant angry faces as stop-signal impair response inhibition compared to happy faces. We interpret these results as a shared common pool of attentional resources between (a) the processing of emotion and the inhibition process and (b) the go process (response execution) and stop process (response inhibition). Overall, these results have three key theoretical implications. First, our results support the “dual competition framework”. More specifically, emotion in the go signal already consumes most of the available resources. In contrast, a neutral go signal consumes fewer resources leaving enough resources for the emotional information of the stop signal to modulate inhibition. Second, our results elucidate the difference in the processing of angry and happy faces in the context of response inhibition; angry faces as go and stop-signal interfered with inhibitory control compared to a happy face. Third, our results further show that the go and stop processes may not be fully independent such that go process is differently decelerated by different emotional information in the stop signal. Thus, our results are better explained by the cancellable-rise-to-threshold (CRTT) model that proposes interaction between go and stop processes. Our results support recent literature that the go and stop processes interact with each other, relying on the same pool of available attentional resources. Since this is the first study where irrelevant emotions were manipulated for both go and stop signals; therefore, further studies are required to ascertain the present study results. Future work should study response inhibition with other emotions such as surprise, disgust, and fear in similar paradigms.

## Data Availability

The datasets generated and/or analysed during the current study are available in the OSF repository, https://osf.io/x52pj/?view_only=6e33aaed2f62458cb13f9cb1da75ba21.
